# Near death experience: neuroscience perspective

**DOI:** 10.3325/cmj.2015.56.392

**Published:** 2015-08

**Authors:** Lukas M. Konopka

**Affiliations:** Department of Psychiatry, Loyola Medical Center, Maywood and Chicago Brain Institute, Rolling Meadows, IL, USA *lmkonopka@gmail.com*

The cycle of life starts with conception and progresses to birth, maturity, old age, and death. We know a significant amount about what happens during most of these stages; however, the final step, death, defies many of our attempts to study and comprehend it. The cultural rituals that surround death attest to our fascination with this concluding stage of life. Cultures often look at death as a transitional stage that sends us on to the next stage of existence, a new reality and consciousness (1). As such, it is only natural that we have significant interest in near-death experiences, where individuals who experienced life-threatening situations report identifiable features of their dying process.

Many such events have been reported by patients who have survived life-threatening situations such as cardiac arrest, trauma, suicide, surgery, etc. One is tempted to hypothesize that age, gender, and culture may influence these experiences (1). Systematic studies of near-death events are difficult; but recently, self-report scales have revealed common features such as out-of-body experiences with euphoria and mystical elements, where the patient hears voices and sounds, passes through a dark space, and often sees a bright light. In addition, some individuals have undergone a rapid life-in-review. Some individuals also describe meeting familiar and unfamiliar people that they identify as mystical or supreme entities. Although In most cases of near-death, people generally report experiences of peace, serenity, and even acceptance, infrequently there have been reports of negative encounters with tormentors and perceptions of Hell (2,3).

So, what can these occurrences tell us about what happens in the brain during the process of death? We know that death often follows acute situations that can influence brain function, eg, cardiac arrest, general anesthesia, and some sleep abnormalities. Imaging studies on cardiac patients who survive near-death experiences, have shown damage in both gray and white matter without brainstem impact (4,5). Apparently, these and other studies reveal common areas involved in the near-death experiences that include the occipital cortex, frontal lobes, hippocampus, basal ganglia, amygdala, and, often, the temporal/parietal junction (6). When the brain undergoes decreased oxygenation, it may react in ways that culminate in a patient’s near-death experience. Other factors such as general anesthesia and substances such as ketamine, LSD, and cannabinoids may provide experiences of joy, visual hallucinations, tunnel vision, and transcendental feelings (7). Patients with abnormal temporal lobe EEG patterns can report a deepening of emotions and a sense of personal destiny (8). When the temporal lobes are stimulated, patients also report memory flashbacks, life-in-review, and experiences of a mystical presence. Out-of-body experiences are primarily associated with the right posterior temporal lobe and the temporal/parietal region (9). Thus, it appears that near-death experiences have common features with other situations and are sometimes induced by pharmacological agents, epileptic discharges, and direct brain stimulation. Nevertheless, there also are well-designed prospective studies on patients who have had a complete loss of brain function yet report near-death experiences despite presumably eliminating the above-described physiological brain events. Even patients whose EEGs have flat-lined have had life-changing experiences. Moreover, studies on blind individuals who perceived visual images during near-death experiences support the notion of perception without a physiological substrate (10). Clearly, these experiences are extraordinary (11). Beyond a doubt, it is scientifically challenging to ask whether these near-death events are perceptions of what lies beyond our normal sensorium and understanding of consciousness (12,13). To understand the abundance of near-death experiences in patients that apparently lack a functioning brain, we may need to step out of our currently accepted physiological paradigms to embrace other explanations of the near-death phenomenon (14). Perhaps, near-death experiences open a window to the concept of a universal consciousness that is free of time and space. The principles of quantum physics provide us the conceptual framework and tools for exploring the ubiquitous experience of death (15).
